# Predicting factors and outcomes of acute myocarditis in children – a 5-year experience in a teaching hospital from the Eastern province of Saudi Arabia

**DOI:** 10.25122/jml-2022-0123

**Published:** 2022-10

**Authors:** Waleed Albuali

**Affiliations:** 1 Department of Pediatrics, College of Medicine, King Fahad Hospital, Imam Abdulrahman Bin Faisal University, Dammam, Kingdom of Saudi Arabia

**Keywords:** myocarditis, pediatric, biomarker, outcome, predictor

## Abstract

Myocarditis in children is not very common, but the state of the disease is similar to viral influenza, making it challenging for a clinician to make an early diagnosis and treatment plan. Elevated levels of inflammatory biomarkers like C-reactive protein, serum lactate, troponin, and abnormal ECG may be helpful for high-index suspicion of myocarditis. This study aimed to determine the predicting factors and outcomes of acute myocarditis in children admitted to a teaching hospital in the Eastern province of Saudi Arabia. Complete medical records of 80 pediatric patients with acute myocarditis over 5 years (from 2015 to 2019) were retrieved, including demographic characteristics, laboratory investigations including cardiac enzymes (serum lactate, CPR, and troponin), and ECG findings for the diagnosis of myocarditis based on tachycardia and bradycardia. There were 22 (27.5%) mortalities that took place during the hospital stay. Low WBC (mmol/dl) levels were associated with in-hospital mortality (p 0.001), whereas high serum lactate levels (>2 mmol) were not. Association of troponin level (0.15ng/mL) was not significantly associated with mortality (p=0.496). The area under the ROC=0.947 (95% C.I: 0.88–1.0) revealed highly significant predictive validity at the 2.3 best cutoff point, having 90.9% and 87.9% sensitivity and specificity, respectively. Clinical factors and elevated biomarkers were associated with poor prognosis and in-hospital mortality in pediatric myocarditis. Serum lactate and troponin levels demonstrated high predictive validity for early diagnosis.

## INTRODUCTION

Myocarditis in children is not very common, but the state of the disease is similar to that of viral influenza. Most of the symptoms overlap as there is a broad spectrum of disease conditions that make it challenging for a clinician to make an early diagnosis and treatment plan. The outcome of myocarditis was also observed with significant variation in relation to multiple clinical factors at presentation, diagnosis, and early treatment response [1–3]. In the general population, almost all age groups are likely to be affected by myocarditis. Still, most infants and adolescents were observed with myocarditis (symptoms include breathing difficulties, chest pain, fainting, fever and rapid breathing), even though more than half of the children were in their first year of life. The main causes of pediatric myocarditis are viruses, including the influenza virus, coxsackie virus, adenovirus, and parvovirus, that reach the heart muscle and damage it, known as the myocardium. However, there may be bacterial causes like Lyme disease [4]. Myocarditis is commonly associated with abnormalities in electrocardiograms (ECG), noninvasive cardiac imaging, and cardiac biomarkers [5]. Myocarditis is usually found in patients with high C-reactive protein, serum lactate, and troponin T, but these markers have a low negative predictive value to rule out patients with other diseases suspected to be myocarditis [5, 6]. Troponin T, troponin I, and C are protein-type cardiac enzymes to control muscle contraction [7], which are observed to be elevated in children with myocarditis [7]. An electrocardiogram (ECG), echocardiography, and possibly an MRI should be included in a noninvasive imaging exam. [8]. The extent of diagnostic accuracy from the validation studies may be a supportive tool by correlating precipitating symptoms and clinical assessment of myocarditis [9]. An abnormal ECG finding is considered 100% sensitive for detecting myocarditis, but a normal ECG is nonspecific to rule out suspected myocarditis based on laboratory findings and presenting symptoms [10]. Myocarditis has been associated with up to 20% of case fatalities and causes sudden infant death syndrome [11, 12]. Based on limited validation studies, we have yet to reach an agreement that precipitating symptoms, elevated cardiac enzymes, and abnormal ECG can be relied on to identify myocarditis, as a late diagnosis can result in significant morbidity and mortality [13]. There are no data available on the exact incidence of myocarditis in the Saudi pediatric population, and all available data is estimated from adult research. Only one single-center retrospective investigation found that the majority of deaths from primary pediatric cardiac illness occur during the first year of life, with a three-year survival rate of 78%. This study was designed to determine the predicting factors and outcomes of acute myocarditis in children admitted to a teaching hospital in the eastern region of Saudi Arabia.

## MATERIAL AND METHODS

### Patient's characteristics

This cross-sectional study included the complete medical records of 80 pediatric patients with acute myocarditis over 5 years (from 2015 to 2019) to evaluate the predictive validity of biomarkers in diagnosing acute myocarditis. Only pediatric patients with acute myocarditis were included, as were poor prognostic indicators such as serum lactate, CPR, troponin, and ECG. Those with acceptable biomarkers were excluded. Ethical approval from the internal review board of the King Fahd hospital, Dammam, Saudi Arabia, was obtained before the commencement of the study, including demographic characteristics, precipitating symptoms, laboratory investigations including cardiac enzymes (serum lactate, CPR, and troponin), and ECG findings for the diagnosis of myocarditis based on tachycardia, typically known as a mean heart rate >2 SD above normal for age, and bradycardia, which is defined as a mean heart rate in the 10^th^ percentile for age, were also documented. The reference value of biomarkers was as follows [14]:

1. 0.2–2.0 mmol/L (plasma venous), >2 mmol/L (elevated);
2. CRP level: 1 mg/ml (normal), 1 mg/ml (elevated);
3. Troponin: 0.15 ng/ml (normal), 0.15 ng/mL (elevated).

### Statistical analysis

Data analysis was performed using the Statistical Package for the Social Sciences (SPSS IBM version 26). All categorical variables, including gender, ECG findings, in-hospital outcome, and predicting factors of myocarditis, were presented as frequencies and percentages. A Chi-square test was used to compare the proportions of categorical variables in relation to expired *versus* survived outcomes. Logistic regression analysis was used to evaluate predictors of myocarditis using the in-hospital outcome as a binary variable. Receptive operating curve (ROC) analysis was performed to calculate the predictive validity of cardiac enzymes, including lactate level and troponin levels, for the patient's outcome and prognosis. Statistically significant results were considered if the P-value was 0.05.

## RESULTS

For 5 years, a total of 80 children with myocarditis were admitted, 58 (72.5%) males and 22 (27.5%) females. The average age of children was 6.04 (ranging from 0 to 14) years. There were 22 (27.5%) mortalities that occurred during the hospital stay, whereas 58 (72.5%) children survived and were successfully discharged. The in-hospital outcome was taken as a binary variable in the logistic regression model, whereas clinical factors and biochemical markers were taken as co-variables to evaluate the predictors of the patient's outcome and prognosis of myocarditis.

**Figure 1:**
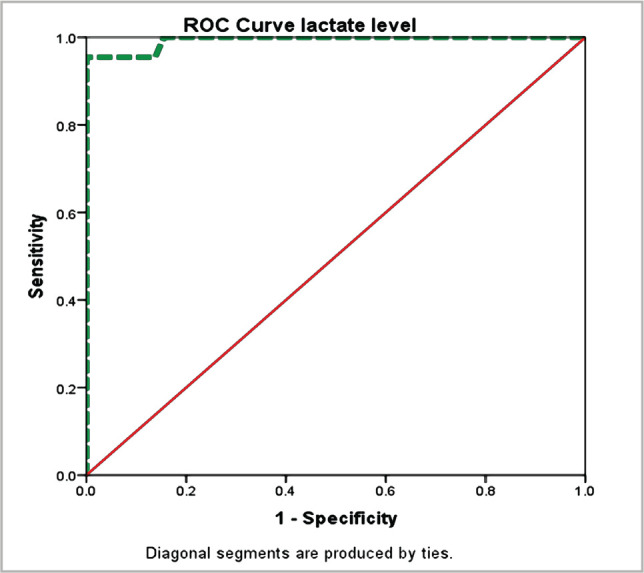
Predictive value of serum lactate level for in-hospital outcome: Area under the curve AUC=0.993 (95% C.I: 0.979–1.0) revealed significantly high predictive validity of serum lactate level with the in-hospital outcome (p<0.001).

**Figure 2:**
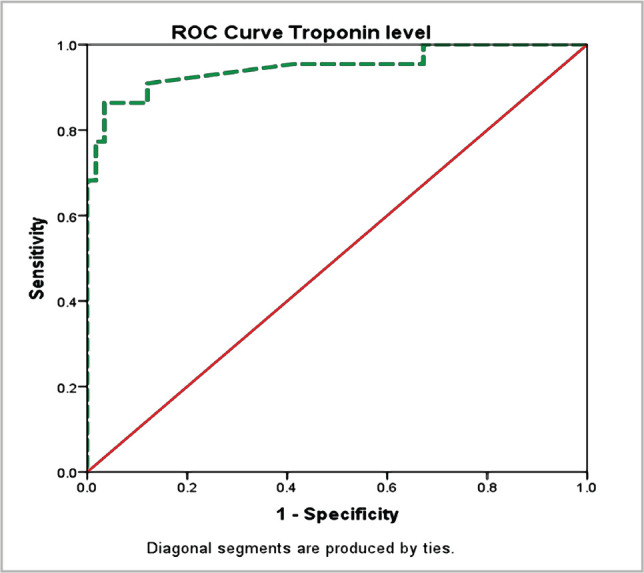
Predictive value of serum troponin level for in-hospital outcome: Area under the curve AUC=0.947 (95% C.I: 0.88–1.0) revealed significantly high predictive validity of serum troponin level with the in-hospital outcome (p<0.001).

The PRISM score was developed to reduce the time it takes to measure the mortality rate for measuring PICU quality. It includes a 12-hour prediction system in addition to a 24-hour prediction method. The PRISM III score >8 cutoff values showed a significant association with in-hospital mortality as approximately 95.5% of myocarditis mortalities were observed in children with PRISM III >8 (p=0.001), whereas 100% survived with normal PRISM III 8. A typically low white blood cell (WBC) count (mmol/dl) significantly affected mortality (p=0.001). A high level of CPR (1 mg/mL) was 23 times more likely to predict in-hospital mortality than a low level of CPR (1 mg/mL). In addition, high serum lactate levels (>2 mmol) were also significantly related to in-hospital mortality (p=0.001). As detailed in Table 1, severe M acidosis was 5 times more likely to cause in-hospital mortality as compared to moderate to mild levels (p=0.004). Clinical factors like sepsis, acute kidney injury, multiple organ failure (MOF), fluid overload, and the number of inotropes needed were also significant contributory factors (Table 2). The predictive validity of cardiac enzymes was evaluated by ROC analysis. As illustrated in Figure 1, the area under the curve AUC=0.993 (95% C.I: 0.979–1.0) revealed significantly high predictive validity of serum lactate level with the in-hospital outcome (p=0.001). The best cutoff point for the highest predictive validity of serum lactate level was 2.1, where sensitivity and specificity of lactate level to predict poor hospital outcomes were 100% and 80%. Figure 2 shows that the area under the curve (AUC)=0.947 (95% CI: 0.88–1.01) revealed a significantly high predictive validity of serum troponin level with the in-hospital outcome (p=0.001). The best troponin level cutoff for predicting poor in-hospital outcomes was 2.3, where sensitivity and specificity were 90.9% and 87.9%, respectively.

**Table 1:** Predicting biomarkers of in-hospital outcome of acute myocarditis.

Biomarkers	Total (n=80) n (%)	Dead (n=22) n (%)	Survived (n=58) n (%)	OR (95% C.I)	P-value
**PRISM score**					
High >8	21 (26.2)	21 (95.5)	0 (0)	1677 (65–4760)	<0.001
Normal ≤8	59 (73.8)	1 (4.5)	58 (100)		
**WBC (X103/µL)**					
Low (<5)	6 (7.5)	6 (27.3)	0 (0)	46.1 (2.5–861)	<0.001
Normal (5.0–14.5)	74 (92.5)	16 (72.7)	58 (100)		
**C-reactive protein**					
High (≥1mg/ml)	22 (27.5)	16 (72.7)	6 (10.3)	23.1 (6.5–81.7)	<0.001
Normal (<1mg/ml)	58 (72.5)	6 (27.3)	52 (89.7)		
**Serum lactate level**					
High (>2 mmol)	43 (53.8)	22 (100)	15 (25.9)	38.9 (4.9–310)	<0.001
Normal (≤2 mmol)	37 (46.2)	0 (0)	43 (74.1)		
**M Acidosis**					
Severe	21 (26.2)	11 (50.0)	10 (17.2)	4.8 (1.6–14.1)	0.004
Up to moderate	59 (73.8)	11 (50.0)	48 (72.8)		
**Troponin level**					
High (>0.15 ng/mL)	77 (96.2)	22 (100)	55 (94.8)	2.8 (0.1–57.2)	0.496
Normal (≤0.15 ng/mL)	3 (3.8)	0 (0)	3 (5.2)		

P-value≤0.05 was considered as statistically significant; WBC – white blood cells.

**Table 2:** Association of clinical factors and in-hospital outcome of acute myocarditis.

Factors	Total (n=80) n (%)	Dead (n=22) n (%)	Survived (n=58) n (%)	OR (95% C.I)	P-value
**Sepsis**					
Yes	19 (27.5)	12 (54.5)	7 (12.1)	8.7 (2.8–27.7)	<0.001
No	61 (72.5)	10 (45.5)	51 (87.9)		
**AKI**					
Yes	36 (45.0)	14 (63.6)	22 (37.9)	2.9 (1.0–7.9)	0.039
No	44 (55.0)	8 (36.4)	36 (62.1)		
**MOF**					
Yes	42 (52.5)	22 (100)	20 (34.5)	84.5 (4.9–1466)	<0.001
No	38 (47.5)	0 (0)	38 (65.5)		
**Fluid overload**					
Yes	33 (41.2)	15 (68.2)	18 (31.0)	4.8 (1.7–13.7)	0.003
No	47 (58.8)	7 (31.8)	40 (69.0)		
**Number of inotropes**					
High (>0.4)	22 (27.5)	14 (63.6)	8 (13.8)	10.9 (3.5–34.4)	<0.001
Normal (0–0.4)	58 (72.5)	8 (36.4)	50 (86.2)		

P-value≤0.05 was considered as statistically significant; AKI – Acute kidney injury; MOF – fluid overload.

## DISCUSSION

The majority of deaths from primary pediatric cardiac illness occur during the first year of life, according to a single-center, retrospective study. As a result, the current research was carried out to determine risk factors and outcomes of acute myocarditis in children admitted to a teaching hospital in Saudi Arabia's Eastern Region. Results from our study demonstrate that typically low WBC (mmol/dl) showed a significant effect on mortality (p=0.001). Still, high serum lactate level (>2 mmol) was significantly related to in-hospital mortality (p=0.001), whereas troponin level was remarkably elevated but had no correlation with in-hospital mortality (p=0.496). During the hospital stay, non-survivors had a higher peak lactate level than survivors, 10 *vs*. 3 mmol/L, which was significant at the 5% level [15]. In a therapeutic study, the AUC of pre-ECMO lactate was 0.848 (95% CI, 0.697–0.999, p 0.002). A pre-ECMO lactate level of 79.8 mg/dL and post-ECMO were appropriate cutoff points to predict mortality [16]. For comparison, there is very limited literature available for comparison of the predictive validity of serum lactate levels in pediatric myocarditis. Troponin levels, on the other hand, are thought to be a reliable indicator [5, 17]. Rady HI et al. reported 100% positive predictive value and specificity of cardiac troponin on 2.02 folds due to multiple laboratory tests and variant normal ranges, whereas they reported 88.7% negative predictive value and 62.5% sensitivity [18]. The area under the curve (AUC) was 0.936 (95% confidence interval (CI): 0.85–1.0, p=0.0001) [18]. It revealed the remarkable power of our study as we had a standard laboratory facility in our set-up and found AUC=0.947 (95% C.I: 0.88–1.0), which exhibits significantly high predictive validity of serum troponin level in relation to the severity of illness (p=0.001), also consistent with AUC (0.96) in another study [19]. Contrarily, Vashist S et al. [20] reported very low sensitivity but high specificity of troponin level, with 34% and 89%, respectively. However, consistent with our study, Sachdeva A et al. [21] exhibited a significant association of elevated troponin level (1) with a poor outcome (p=0.04). Rodriguez-Gonzalez M et al. did not find any relationship between troponin level (p=0.789) and CRP (p=0.443) with poor outcome, but the sample size in this therapeutic study was 42, while our sample size was double [9]. Our study provides sufficient evidence to support the statement that cardiac troponin was mainly elevated in patients with acute, early-onset myocarditis, whereas the absence of increased levels suggests a long-term presence of myocarditis [22]. At admission, WBC was 11.47.1×103/ul, CRP was 0.650.16 mg/dl, and troponin was 0.410.19 ng/ml in one study [23]. However, due to higher variability, the mean does not represent the true picture, therefore, we categorized the numeric response variable as per standard normal ranges. Chong SL et al. derived a risk score of a maximum of five scores based on five domains of presenting characteristics, including vital signs, physical examination, CXR and ECG abnormalities. Although they estimated the area under the ROC at 0.90 (95% CI: 0.83 to 0.97), the process of risk scoring was complex and time-consuming to rule out misdiagnosis and initiate appropriate treatment [10]. The results of our study may be encouraging for clinicians to make rapid provisional diagnoses based on biomarkers taken on bedside blood samples at presentation and rule out misdiagnosis following the results of biomarkers at regular intervals and other diagnostic procedures. It is recommended to design more prospective studies by taking confirmatory gold-standard criteria to validate highly suspected acute myocarditis based on biomarkers at periodic intervals and best cutoff points.

## CONCLUSION

Clinical factors and elevated biomarkers were associated with poor prognosis and in-hospital mortality in pediatric myocarditis. Serum lactate and troponin levels have demonstrated high predictive validity for early diagnosis. A combined approach of regular monitoring of biomarkers and ECG findings may be helpful in the initiation of appropriate treatment and improve the patient's outcome.

## Data Availability

Further data is available from the corresponding author on reasonable request.
